# Improving working memory in children with low language abilities

**DOI:** 10.3389/fpsyg.2015.00519

**Published:** 2015-04-30

**Authors:** Joni Holmes, Sally Butterfield, Francesca Cormack, Anita van Loenhoud, Leanne Ruggero, Linda Kashikar, Susan Gathercole

**Affiliations:** ^1^Medical Research Council Cognition and Brain Sciences Unit, CambridgeUK; ^2^Cambridge Cognition, CambridgeUK; ^3^Cognitive Psychology Unit, Department of Psychology, Leiden University, LeidenNetherlands; ^4^Speech and Language Therapy Department, Davison Therapy Centre, Brookfields Hospital, CambridgeUK; ^5^Georg-Elias-Müller-Institut für Psychologie, University of Göttingen, GöttingenGermany

**Keywords:** working memory, SLI, language, intervention, cognitive training, verbal IQ

## Abstract

This study investigated whether working memory training is effective in enhancing verbal memory in children with low language abilities (LLA). Cogmed Working Memory Training was completed by a community sample of children aged 8–11 years with LLA and a comparison group with matched non-verbal abilities and age-typical language performance. Short-term memory (STM), working memory, language, and IQ were assessed before and after training. Significant and equivalent post-training gains were found in visuo-spatial short-term memory in both groups. Exploratory analyses across the sample established that low verbal IQ scores were strongly and highly specifically associated with greater gains in verbal STM, and that children with higher verbal IQs made greater gains in visuo-spatial short-term memory following training. This provides preliminary evidence that intensive working memory training may be effective for enhancing the weakest aspects of STM in children with low verbal abilities, and may also be of value in developing compensatory strategies.

## Introduction

Impairments in working memory are common in many developmental disorders (Martinussen et al., 2005; Carretti et al., 2009) and have been suggested to act as barriers to educational achievement (Swanson and Sachse-Lee, 2001; Gathercole and Alloway, 2006, 2008; Archibald and Joanisse, 2009). This has led to widespread interest in the possibility that the working memory abilities of children who are poor learners could be enhanced through intensive training in memory-taxing activities. In both children with attention deficit hyperactivity disorder (ADHD) and those with low working memory alone, Cogmed working memory training (Cogmed, 2005) enhances performance on untrained measures of working memory (e.g., Klingberg et al., 2005; Holmes et al., 2010; Chacko et al., 2013; Dunning et al., 2013). Benefits of training have also been reported in other developmental populations including survivors of pediatric cancer with poor working memory (Hardy et al., 2011) and typically developing preschool children (Thorell et al., 2009). The novel issue addressed by the present study is whether the benefits of working memory training are modulated by the language-related abilities of the trainees.

Working memory provides the temporary storage of information needed to guide ongoing cognitive activities. A variety of models of working memory have been advanced that vary widely in their specificity and scope (Unsworth and Engle, 2007; Cowan, 2010; Oberauer et al., 2012). The multi-component model developed originally by Baddeley and Hitch (1974) and elaborated by Baddeley (2000) has proved to be a particularly useful framework for characterizing the development of working memory during childhood (Bayliss et al., 2005; Alloway et al., 2006; Henry, 2011). The model consists of a central executive that controls the allocation of attentional resources required to maintain information in working memory. This is supplemented by specialized limited-capacity stores that maintain verbal and visuo-spatial information, and an episodic buffer that integrates multi-modal representations within working memory. Two broad classes of test assess the different components of this model. STM tasks involve the simple recall or recognition of information in the form in which it was presented, and assess the capacity of either the verbal or visuo-spatial store according to the domain of the stored information. Examples of STM paradigms are digit span (verbal) and block span (visuo-spatial). The central executive is often assessed by complex span tasks imposing significant processing as well as storage. Examples include backward digit span (the recall of digits in reverse sequence) and Mr. X (a visuo-spatial task involving spatial comparisons of two images and the retention of sequences of spatial information, Alloway et al., 2006).

Cogmed training has been suggested to improve the neural efficiency of the brain networks involved in working memory through intensive practice (Westerberg and Klingberg, 2007; Karbach and Schubert, 2013; Astle et al., 2015). It has also been identified as a potential solution to developmental impairments of working memory problems (Klingberg, 2010; Sonuga-Barke et al., 2013). In the present study, we investigated whether Cogmed training can overcome the working memory problems typically found in children with low language learning abilities. Children diagnosed with Specific Language Impairment (SLI), a condition characterized by poor language learning in the absence of general intellectual problems, have been widely reported to have deficits on measures of both verbal STM and verbal complex memory span (Montgomery, 1995; Bishop et al., 1999; Botting and Conti-Ramsden, 2001; Archibald and Gathercole, 2006). In contrast, their performance on visuo-spatial memory tasks is appropriate for their age (Bavin et al., 2005; Archibald and Gathercole, 2006). A similar profile of predominantly verbal impairments in working memory is also present in children with reading difficulties (Catts et al., 2002; Pickering, 2006).

It has been proposed that deficits in the phonological loop may underlie some of the language learning problems of children with SLI (Baddeley et al., 1998; Archibald and Gathercole, 2007). However, the more widely accepted view is that developmental impairments of language such as SLI arise from a core deficit in phonological coding which impacts on any activities (including memory tasks) with significant demands on the quality of phonological representations (de Jong, 1998; Bishop and Snowling, 2004; Catts et al., 2006). These two views generate conflicting hypotheses regarding the impact of training on children who are poor language learners. A deficit that originates in the phonological loop may be compensated for either directly by improvements in the efficiency of the working memory neural substrate resulting from intensive adaptive training (Klingberg, 2010) or more indirectly from improved strategy use (Dunning and Holmes, 2014). Alternatively, if the core deficit is in phonological coding and the temporary storage problems for verbal materials are therefore downstream from this, training that taxes STM and working memory would not be expected to ameliorate the continuing encoding deficit. On this basis it is predicted that children with poor language would have a diminished response to training on verbal memory tasks compared with individuals with typical language abilities. The aim of the present study was to test these contrasting hypotheses.

A variety of memory training programs exist (e.g., N-back training; Jaeggi et al., 2008), but the one most widely used in research studies with children is Cogmed Working Memory Training, which involves training for 25 days on a variety of memory-taxing activities employing both visuo-spatial and verbal materials. It has been applied across many studies to populations with domain-general deficits in STM and working memory, including children with ADHD and those with working memory problems in the absence of a diagnosed attentional deficit. In these groups, the benefits of training extend across untrained verbal and visuo-spatial complex memory tasks (Klingberg et al., 2005; Holmes et al., 2009, 2010; Gray et al., 2012; Chacko et al., 2013; Dunning et al., 2013; Rapport et al., 2013) and visuo-spatial STM tasks (Klingberg et al., 2005; Holmes et al., 2010; Dunning et al., 2013). Training benefits for verbal STM are less consistent. They are present in some children (Klingberg et al., 2002, 2005; Holmes et al., 2009) but not in others (Holmes et al., 2009; Gray et al., 2012; Dunning et al., 2013). Differences in the transfer tests employed across studies may contribute to these inconsistencies.

The impact of Cogmed training was compared between children with poor language abilities (LLA) and a comparison group of children with age-appropriate language that were matched on non-verbal IQ. This design is appropriate for investigating differential responses to training across groups, but not for quantifying the highly specific benefits of a particular training program due to the absence of active or passive intervention conditions. Members of the LLA group were selected through community screening on measures of both an expressive language (sentence repetition) and a receptive language (picture-word matching) test. None of the children were diagnosed with language impairments (although their problems had in many cases been recognized by their schools) but their language profile corresponds closely to that of children with SLI and related language learning problems meaning the results will nonetheless be relevant to this group too (e.g., Bishop et al., 2000; Conti-Ramsden et al., 2001). However, the standard SLI exclusionary criterion of a marked discrepancy between language and non-verbal abilities was not applied (Bishop et al., 1999; Tomblin and Zhang, 1999). The reason for this is that because working memory and fluid intelligence are known to be closely linked (Engle et al., 1999; Jaeggi et al., 2008), the exclusion of low scorers could potentially eliminate individuals with working memory problems. The current selection approach also enabled us to evaluate the extent to which Cogmed training was beneficial for a sample that were more representative of the majority of poor language learners in school than children with a diagnosis of SLI.

A secondary aim of the study was to investigate whether the children’s responses to training on measures of working memory were mediated by a broader range of individual differences in their cognitive abilities other than the selection measures of language. To provide the necessary power for these exploratory correlational analyses, data from both groups was combined. While no specific hypotheses were generated, it was speculated that pre-training strengths in working memory may support the development of new and possibly compensatory strategies through training (Dunning and Holmes, 2014). Support for this would be provided if high baseline memory performance was associated with greater training gains on the working memory transfer tests.

## Materials and Methods

### Participants

A total of 179 children aged 8–10 years attending two primary schools in south–east England were screened on a receptive language test [Peabody Picture Vocabulary Test (PPVT), Dunn and Dunn, 2007], an expressive language test [Recalling Sentences subtest of the Clinical Evaluation of Language Fundamentals (CELF), Semel et al., 2006], and a test of non-verbal reasoning [Matrix Reasoning subtest of the Wechsler Abbreviated Scales of Intelligence (WASI), Wechsler, 1999]. All children were native English speakers (87 males, mean age 9 years, 3 months, SD = 10.7 months). Of the screened sample, 16 children with standard scores <86 on PPVT and scaled scores <7 on CELF Recalling Sentences formed the LLA group. A comparison group of 16 children were individually matched on age to within 90 days, gender, and on non-verbal reasoning. The two groups differed on CELF Recalling Sentences, *t*(30) = -10.692, *p* < 0.001, *d* = 3.784, and on PPVT, *t*(30) = -7.69, *p* < 0.001, *d* = 2.987. There were no group differences on the non-verbal reasoning task, *t*(30) = -0.244, *p* = 0.809, *d* = 0.086. Both groups scored in the low average range on this task.

Consent to continue to the training phase was not obtained for one child in the LLA group, and two further children (one in each group) withdrew before any further testing was completed. Two children in the LLA group failed to complete training (one withdrew and the other moved schools). Data are reported here only for the remaining children who completed training (LLA, *n* = 12, males = 7, mean age 9 years, 9 months, SD = 8.4 months; comparison group, *n* = 15, males = 8, mean age 9 years, 9 months, SD = 9.5 months). Descriptive statistics for the screened sample and both groups are shown in **Table 1**. The LLA group scored at a significantly lower level on the Recalling Sentences test, *t*(25) = -11.687, *p* < 0.001, *d* = 4.513 and the PPVT, *t*(25) = -6.613, *p* < 0.001, *d* = 2.938, with no significant group differences in non-verbal reasoning, *t*(25) = -0.503, *p* = 0.619, *d* = 0.194.

**Table 1:** Language and non-verbal reasoning profiles of screening sample and selected groups.

		CELF Recalling Sentences		Peabody Picture Vocabulary Test (PPVT) vocabulary		Matrix Reasoning	
	*n*	*M*	SD	*M*	SD	*M*	SD
Screening sample	179	9.220	3.170	100.630	13.600	47.280	10.120
LLA	12	3.750	1.603	81.083	4.078	41.250	6.917
Comparison	15	10.800	1.521	103.067	10.886	42.600	6.936

### Procedure

Following screening, participants completed a set of pre-training assessments in a one-to-one session that lasted approximately 1.5 h. They then took part in 20 45-min sessions of Cogmed Working Memory Training over the following 8 weeks in small groups in school supervised by a research assistant. Upon completion of training, all pre-training tasks were re-administered in individual sessions. The researchers conducting the pre- and post-training assessments and supervising training were blind to group membership.

#### Working Memory

Participants completed eight subtests of the Automated Working Memory Assessment (AWMA, Alloway, 2007) before and after training: two tests each of verbal STM (Digit Recall, Word Recall), visuo-spatial STM (Dot Matrix, Block Recall), verbal working memory (Backward Digit Recall, Listening Recall), and visuo-spatial working memory (Mr. X, Spatial Recall). The verbal STM and working memory tasks required spoken responses. Pointing responses were required for the visuo-spatial tasks. The STM tasks required the immediate serial recall of either verbal or visuo-spatial information (e.g., recalling a digit list in forward order). The working memory tasks had an additional executive load in the form of processing either the storage material or other relevant information prior to recall (e.g., reversing digit sequences prior to recall). Standard scores were derived for individual tests. Composite scores for each of the four aspects of working memory were calculated by averaging standard scores for each pair of tests.

#### Language

At the pre-training assessment, participants completed a test of phonological processing, and verbal STM the Children’s Test of Non-word Repetition (CNRep, Gathercole and Baddeley, 1996), and the Understanding Spoken Paragraphs subtest of the CELF (Semel et al., 2006), a measure of listening comprehension. The same assessments were completed again after training, in addition to the PPVT (Dunn and Dunn, 2007) and CELF Recalling Sentences tests.

#### IQ

Prior to training, the Similarities and Vocabulary (verbal IQ), and Block Design (performance IQ) subtests of the WASI (Wechsler, 1999) were administered. The fourth subtest, Matrix Reasoning, was administered at screening. All four measures of the WASI were administered after training. Composite indices of verbal and performance IQ were calculated.

#### Working Memory Training

Participants completed 20 sessions of Cogmed Working Memory Training RM (Cogmed, 2005). Each training session lasted approximately 45 min and involved repeated practice on span-like STM and working memory tasks. Participants completed eight out of a possible 12 tasks in each session, with 15 trials on each task. Seven of the tasks involved the serial recall of visuo-spatial information. Of these, four required mental manipulation (e.g., spatial rotation) prior to recall (visuo-spatial working memory) and three required simple serial recall (visuo-spatial STM). Three further tasks required the serial recall of verbal information in the same order (verbal STM) or in reverse or ascending order (verbal working memory). Two other tasks required the recall of verbal information associated with specific spatial locations, one in forward order (STM) and one in reverse sequence (working memory). All responses were made by clicking with the computer mouse. The difficulty of the tasks adapted to match the children’s performance level on a trial-by-trial basis. Full details about the training program are provided at www.cogmed.com/rm.

## Results

### Pre-Training

Descriptive statistics for the STM and working memory tasks are provided in **Table 2**. Language and IQ scores are presented in **Table 3**. Separate multivariate analyses of variance (MANOVAs) were conducted on the STM, working memory, language, and IQ measures. Univariate *F* tests were performed to compare performance between the LLA and comparison groups on the individual measures. Bonferroni corrections were made to correct for multiple testing. Thresholds for statistical significance were *p* < 0.0125 for STM, working memory and IQ, and *p* < 0.006 for language measures.

**Table 2:** Descriptive statistics and group comparisons before and after training for working memory measures.

	LLA group							Comparlson group							Pre-training group differences			Main effect of training			Group x training interaction		
	Pre-training		Post-training		Pre to post			Pre-training		Post-training		Pre to post					
	*M*	SD	*M*	SD	*t*	*p*	*d*	*M*	SD	*M*	SD	*t*	*p*	*d*	*F*	*p*	*d*	*F*	*p*	$\eta_{\rho }^{2}$	*F*	*p*	$\eta_{\rho }^{2}$
**Short-term memory**
Digit Recall	83.875	10.964	90.717	14.474	-3.105	0.010	0.538	107.140	11.014	109.927	17.009	-0.741	0.471	0.199	29.864	0.000	2.117	4.275	0.049	0.146	0.758	0.392	0.029
Word Recall	85.767	12.164	89.008	7.994	-0.910	0.382	0.322	104.167	10.831	106.287	11.008	-0.629	0.539	0.194	17.256	0.000	1.600	1.181	0.287	0.045	0.05	0.822	0.002
Verbal STM	84.821	10.584	89.863	10.415	-2.489	0.030	0.480	105.653	9.174	108.107	11.644	-1.052	0.311	0.236	30.008	0.000	2.109	5.559	0.027	0.182	0.66	0.423	0.026
Dot Matrix	94.075	6.963	106.058	19.822	-2.030	0.067	0.895	93.360	10.736	109.873	17.862	-3.727	0.002	1.155	0.040	0.844	-0.081	15.522	0.001	0.383	0.39	0.537	0.015
Block Recall	89.325	11.931	103.908	17.751	-3.268	0.007	0.983	91.440	8.804	102.520	17.932	-2.608	0.021	0.829	0.281	0.601	0.204	17.099	0.000	0.406	0.32	0.577	0.013
VS STM	91.700	7.287	104.983	17.108	-3.057	0.011	1.089	92.400	7.698	106.197	15.535	-3.988	0.001	1.188	0.058	0.812	0.093	24.420	0.000	0.494	0.01	0.926	0.000
**Working memory**
Listening Recall	93.867	13.890	97.067	7.260	-0.836	0.421	0.303	103.067	13.915	104.793	12.681	-0.396	0.698	0.130	2.919	0.100	0.662	0.682	0.417	0.027	0.06	0.807	0.002
Backward Digit Recall	92.833	9.571	95.842	13.252	-0.855	0.411	0.264	100.400	12.648	108.387	10.825	-2.406	0.030	0.680	2.939	0.099	0.681	5.104	0.033	0.170	1.05	0.316	0.040
Verbal WM	93.350	6.133	96.454	6.986	-1.063	0.311	0.473	101.733	9.885	106.590	10.704	-1.500	0.156	0.472	6.574	0.018	1.047	3.174	0.087	0.113	0.15	0.698	0.006
Mr X	98.250	15.493	98.317	14.453	-0.014	0.989	0.004	98.267	15.248	102.913	10.538	-1.147	0.271	0.360	0.000	0.998	0.001	0.572	0.456	0.022	0.54	0.469	0.021
Spatial Recall	97.542	16.293	101.675	16.670	-1.295	0.222	0.251	102.380	10.044	108.460	12.072	-2.412	0.030	0.550	0.901	0.352	0.367	6.491	0.017	0.206	0.24	0.631	0.009
VS WM	97.896	13.396	99.996	12.544	-0.633	0.539	0.162	100.323	10.963	105.687	9.385	-1.974	0.069	0.527	0.269	0.609	0.199	3.093	0.091	0.110	0.59	0.449	0.023


**Table 3:** Pre and post training scores by group, and group comparisons for language and IQ.

	LLA Group							Comparison group							Pre-training group differences			Main effect of training			Group x training interaction		
	Pre-training		Post training		Pre to post training			Pre-training		Post-training		Pre to post					
	M	SD	*M*	SD	*t*	*p*	*d*	*M*	SD	*M*	SD	*t*	*p*	*d*	*F*	*p*	*d*	*F*	*p*	$\eta_{\rho }^{2}$	*F*	*p*	$\eta_{\rho }^{2}$
**Language**
*CELF*	Recalling Sentences Understanding Spoken	3.750	1.603	3.917	1.881	$-$0.518	0.615	0.096	10.800	1.521	10.600	2.098	0.480	0.638	-0.111	136.583	0.000	4.514	0.004	0.952	0.000	0.448	0.510	0.018
	Paragraphs	5.667	2.807	6.917	2.610	-1.820	0.096	0.462	9.533	1.642	9.867	2.066	0.601	-0.536	0.180	20.013	0.000	1.738	2.190	0.100	0.104	0.975	0.333	0.038
*PPVT*	Vocabulary	81.083	4.078	82.667	9.267	-0.628	0.543	0.237	103.067	10.886	99.133	9.819	1.175	0.260	-0.380	43.730	0.000	2.938	0.288	0.596	0.011	1.589	0.219	0.060
*CN-Rep*	Total	20.417	6.882	29.667	4.250	-5.088	0.000	1.662	31.000	3.684	34.533	2.924	-3.486	0.004	1.069	23.259	0.000	2.003	14.733	0.000	0.626	8.354	0.008	0.250
	2 Syllables	8.500	1.446	9.500	0.674	-1.970	0.074	0.943	9.533	0.915	9.667	0.617	-0.487	0.634	0.174	5.124	0.033	0.875	4.304	0.048	0.147	2.517	0.125	0.091
	3 Syllables	6.167	2.209	8.000	1.206	-2.823	0.017	1.074	7.667	1.345	8.800	1.014	-2.747	0.016	0.961	4.747	0.039	0.844	16.049	0.000	0.391	0.894	0.354	0.035
	4 Syllables	2.833	2.368	5.667	2.387	-4.017	0.002	1.192	6.867	1.767	8.067	1.163	-2.167	0.048	0.819	25.724	0.000	1.951	20.845	0.000	0.455	3.148	0.076	0.120
	5 Syllables	2.917	1.505	6.500	1.883	-6.967	0.000	2.115	6.933	1.223	8.000	1.195	-4.298	0.001	0.882	28.646	0.000	2.945	75.313	0.000	0.751	22.06	0.000	0.469
**IQ**
*WASI*	Verbal IQ	88.333	8.542	91.917	14.625	-1.553	0.149	0.309	103.200	12.143	110.933	8.128	-3.112	0.008	0.763	12.848	0.001	1.437	10.673	0.003	0.299	1.435	0.242	0.054
	Performance IQ	89.083	9.356	92.083	11.333	-2.283	0.043	0.290	89.800	5.480	94.867	8.855	-3.078	0.008	0.707	0.062	0.806	0.097	13.069	0.001	0.352	0.893	0.354	0.034

#### Short-Term Memory

There was a significant group effect on the STM measures, Hotelling’s *T*^2^(4, 22) = 7.497, *p* < 0.001, $\eta_{\rho }^{2}$ = 0.577. Univariate analyses revealed significant group differences on each of the individual verbal STM subtests and the resulting verbal STM composite score, with effect sizes ranging from *d* = 1.6 to 2.12. In all cases, the LLA group scored at a significantly lower level than the matched comparison group. The groups did not differ significantly on the visuo-spatial STM tasks.

#### Working Memory

The group effect was not significant, Hotelling’s *T*^2^(4, 22) = 1.535, *p* = 0.227, $\eta_{\rho }^{2}$ = 0.218. The group difference on the verbal working memory composite score did not withstand the correction for multiple comparisons (*p* = 0.018, *d* = 1.05). Significant group differences were not found either for measures of visuo-spatial working memory or for the individual verbal working memory subtests.

#### Language

A MANOVA revealed a significant group effect for the CELF language tasks, Hotelling’s *T*^2^(2, 24) = 84.943, *p* < 0.001, $\eta_{\rho }^{2}$ = 0.876, and for CN-Rep, Hotelling’s *T*^2^(4, 22) = 17.325, *p* < 0.001, $\eta_{\rho }^{2}$ = 0.759. Total scores for the LLA group were significantly lower than those of the comparison group across all language tasks. On non-word repetition, the LLA group performed significantly more poorly at syllable lengths four and five, with no group difference at shorter syllable lengths.

#### IQ

The group effect for the IQ tests was significant, Hotelling’s *T*^2^(2, 24) = 6.444, *p* = 0.006, $\eta_{\rho }^{2}$ = 0.349. Univariate *F* tests revealed significant group differences in verbal IQ but not in performance IQ. The scores of the LLA group were lower than those of the comparison group on the verbal IQ test.

### Training

Significant main effects of training were observed for the whole sample from pre- to post-test on two visuo-spatial STM tasks, Dot Matrix, Block Recall, and for the derived composite visuo-spatial STM score (see **Table 2**). Scores were higher after training on Digit Recall (*p* = 0.05), Backward Digit Recall (*p* = 0.02), Spatial Recall (*p* = 0.017), and the verbal STM composite score (*p* = 0.03), but in all cases these effects did not meet significance at the Bonferroni threshold. No other main effects for the STM and working memory measures reached significance. Significant gains from pre- to post-test were also observed for the total non-word repetition score and for performance on this test at syllable lengths 3 and 5. There was a main effect of training on both verbal and performance IQ, with significantly higher scores after training (**Table 3**).

Pre- to post-training differences were analyzed separately for each group in paired-sample *t*-tests. Significant increases in scores were observed after training for the comparison group on the following measures: Dot Matrix, Block Recall, visuo-spatial STM, Backward Digit Recall, Spatial Recall, total non-word repetition score, accuracy of repeating 3, 4, and 5 syllable non-words, and both performance IQ and verbal IQ. Significant pre- to post-changes did not withstand the correction for multiple comparisons for some tasks, although the effect sizes were substantial: Block Recall (*d* = 0.829), Backward Digit Recall (*d* = 0.680), Spatial Recall (*d* = 0.550), CN-Rep 3 and 4 syllable non-words (*d* = 0.961 and 819, respectively). This reflects the relatively low statistical power of the study.

For the LLA group, significant gains were found on Digit Recall, verbal STM, Block Recall, visuo-spatial STM, total non-word scores, and performance at syllable lengths 3, 4, and 5, and performance IQ. Gains on the verbal STM composite measure were no longer significant when corrections were made for multiple comparisons, although the effect size was moderate in magnitude, *d* = 0.48. Changes in non-word repetition scores at syllable length 3 did not withstand the multiple comparison correction, but the effect size was large (*d* = 1.074). After correction for multiple comparisons, gains for performance IQ were small (*d* = 0.290) and non-significant.

To investigate group differences in training gains, a series of 2 × 2 ANOVAs with time (pre, post) and group (LLA, comparison) were performed. The outcomes of these analyses are displayed in **Tables 2** and **3**. There were no significant differences in the impacts of training on memory scores in the two groups. Significant group × time interactions were observed for total non-word repetition scores and for performance at syllable length 5. Training gains were significantly greater for the LLA group on both measures. No other training-related differences between groups reached significance. An equivalent pattern of results emerged when group differences in gains scores were compared.

### Correlational Analyses

Initial exploratory correlational analyses were performed between baseline cognitive abilities and gains in STM and working memory on tests that were not used for selection purposes. For these analyses, data from both groups were combined and composite scores derived where there were multiple variables for a single construct in order to reduce error and maximize the case to variable ratio. **Table 4** shows the correlations between measures of IQ, listening comprehension and non-word repetition and gains in each aspect of working memory calculated by the difference between post-training and pre-training scores. Pre-training verbal IQ was highly and negatively correlated with gains in verbal STM (*r* = -0.548), indicating that greatest training benefits were obtained for the children with lowest verbal IQs. There were no other significant associations between pre-training scores and gains in any of the four composite memory scores.

**Table 4:** Correlations between gains in working memory (post minus pre training scores) and baseline ability scores (above horizontal) and between residual training scores in working memory and baseline ability scores (below horizontal).

	Verbal	VS	Verbal	VS	Verbal	Performance	Understanding	CN-Rep
	STM	STM	WM	WM	IQ	IQ	Spoken Paragraphs	Total
Verbal STM	–	0.001	0.343	0.081	-0.548^∗∗^	-0.194	0.003	-0.256
VS STM	0.040	–	0.153	0.471^∗^	0.082	0.357	-0.187	0.163
Verbal WM	0.322	0.270	–	0.056	-0.046	-0.136	0.096	0.059
VS WM	-0.048	0.575^∗∗^	0.265		0.178	0.083	-0.022	0.345
Verbal IQ	-0.382^∗^	0.067	0.208	0.419^∗^	–	0.251	0.446^∗^	0.487^∗^
Performance IQ	-0.172	0.350	-0.039	0.377	0.251	–	-0.171	0.118
Understanding Spoken Paragraphs	0.148	-0.191	0.341	-0.006	0.446^∗^	-0.171	–	0.409^∗^
CN-Rep Total	-0.037	0.149	0.301	0.483^∗^	0.487^∗^	0.118	0.409^∗^	–

^**^denotes significance <0.01, ^*^significant at the <0.05 level.

**FIGURE 1:**
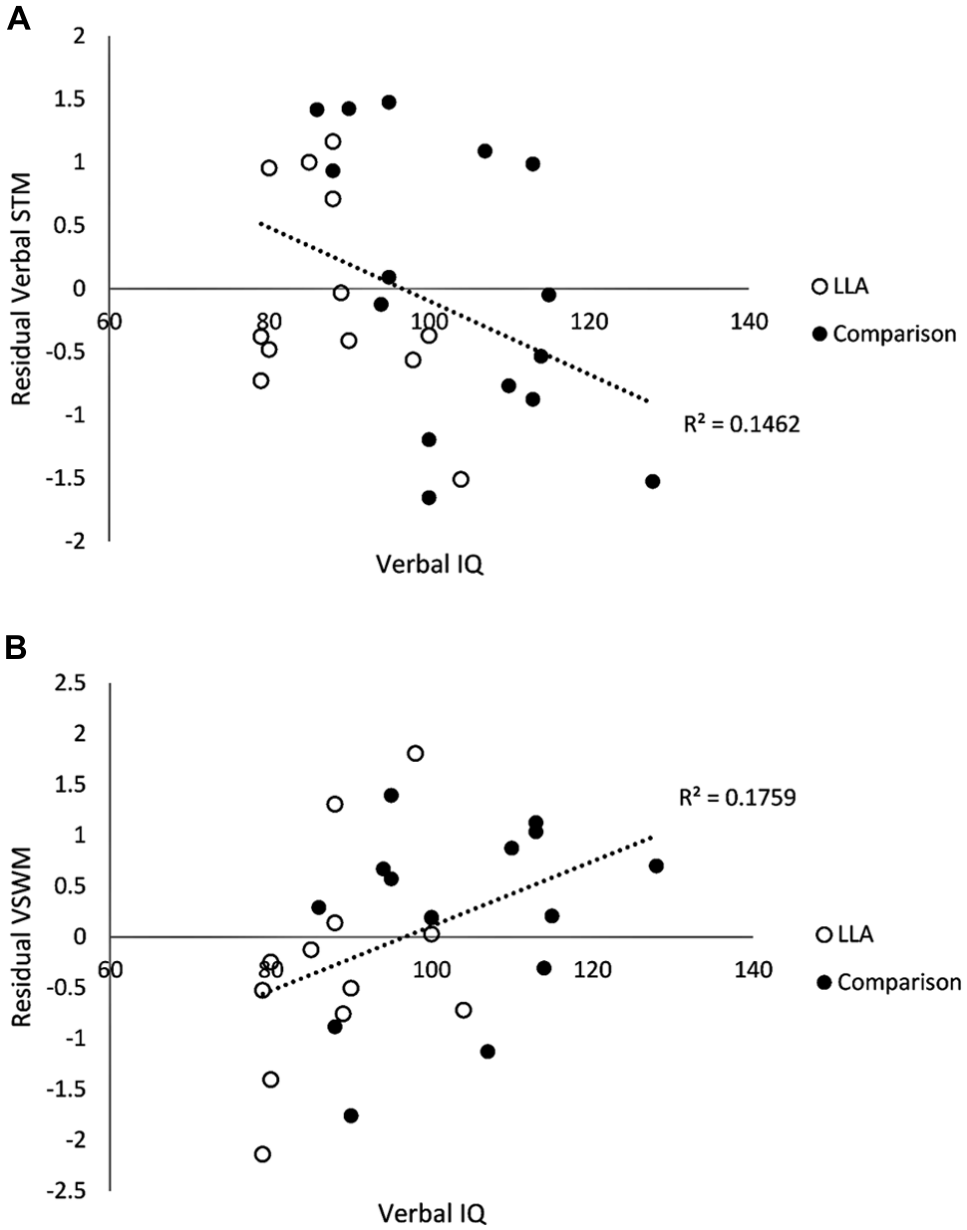
**Scatter plot of baseline verbal IQ and residual training scores in verbal STM **(A)** and visuo-spatial working memory (B)**.

Next, links between pre-training abilities and the variance in post-training working memory scores that could not be predicted by the same working memory assessments taken prior to training were explored. First, the residual variance in post-training scores was calculated from the best-fitting linear function with pre-training scores as the dependent variable, for each of the four working memory composite scores. Correlation coefficients were then calculated between pre-training measures and residual post-training scores. Verbal IQ was significantly negatively associated with the verbal STM residual training score (*r* = -0.382, see **Figure 1A**). This indicates that the relationship between verbal IQ and scores on verbal STM after training was not a simple function of a pre-training association between verbal IQ and verbal STM that had a secondary impact on post-training scores.

Two further significant associations emerged from the analyses of the training residual scores. Post-training residual scores on visuo-spatial working memory were highly associated both with verbal IQ (*r* = 0.419; **Figure 1B**) and non-word repetition (*r* = 0.483).

## Discussion

This study compared the benefits of working memory training for children with LLA and a comparison group with typical language skills. Prior to training, the LLA children scored at relatively low levels on verbal measures of both STM and working memory, and similarly, to the comparison group on visuo-spatial memory tasks. This is consistent with previous reports of close associations between verbal abilities such as vocabulary and verbal STM, both in unselected samples of children (Gathercole et al., 1999; Majerus et al., 2006) and individuals with SLI (Gathercole and Baddeley, 1989; Conti-Ramsden et al., 2001).

The primary aim of the study was to test between predictions derived from contrasting theories of developmental language impairments concerning responses to working memory training of the LLA group. On the basis of the phonological processing deficit account of developmental language impairment (e.g., Bishop and Snowling, 2004), it was predicted that their gains in verbal aspects of working memory would be minimal as the nature of the training program would not be expected to tax input processing skills. In contrast if, like children with SLI, this group have a core deficit in verbal STM (Baddeley et al., 1998; Gathercole, 2006), training that enhances this memory component would be expected to yield significant gains.

A mixed pattern of response to training emerged for verbal STM measures. The comparison group made no gains on any verbal STM measure following training, consistent with findings from studies with other populations including children with low working memory and those with ADHD (Holmes et al., 2009; Gray et al., 2012; Dunning et al., 2013). However, the LLA group improved significantly on one of the two verbal STM measures (digit span, but not word span), although here the training by group interaction was not significant. No strong conclusions can therefore be drawn about whether training has a substantial reliable impact on serial recall measures of verbal STM in the group with LLA.

This group did, however, show a marked differential increase in repetition accuracy for the lengthiest multisyllabic items on the test of non-word repetition relative to the comparison group. The finding is important, as it has been suggested that difficulties in repeating non-words in children with the more severe condition of SLI may reflect underlying verbal STM deficits that also contribute to their vocabulary learning difficulties (Baddeley et al., 1998; Gathercole, 2006). An intervention that targets this ability could therefore have potential for gains in language learning. However, caution is required in interpreting these results in the absence of control training conditions in the present study; as a consequence, training is confounded with repeat testing. The improved accuracy of repeating five-syllable non-words in the LLA group after training (whose pre-training performance was extremely low at 29% compared with 69% for the comparison group) may simply reflect a practice effect rather than a genuine differential benefit of training. This effect was also shown in the comparison group but at a reduced rate, possibly because some of the group’s baseline scores may be close to ceiling. Further studies with randomized allocation of participants to working memory training and suitable control conditions are needed to tease these possibilities apart.

In line with many previous studies (e.g., Melby-Lervåg and Hulme, 2013), both groups made substantial gains on visuo-spatial STM. This outcome is likely to reflect the large number of Cogmed tasks requiring the mental manipulation and storage of visual material. Verbal and performance IQ scores also increased following training for both groups. In the absence a control intervention condition, these improvements at post-assessment are difficult to interpret as they may reflect non-specific features of training such as daily structured engagement and regular feedback rather than the consequences of cognitive improvements following memory-taxing practice. Indeed, evidence from randomized controlled trials has yielded little evidence of selective enhancement of nonverbal reasoning with working memory training (e.g., Redick et al., 2013).

A secondary aim of the study was to investigate whether responses to training are modulated by individual differences prior to training. Exploratory analyses performed on data from the two groups combined to form a single sample revealed some strong predictive links between pre-training scores and training outcomes. First, training improved visuo-spatial working memory to the greatest extent for children with higher verbal abilities. While not a specific a priori prediction, this pattern of findings is broadly consistent with the predictions from the verbal STM account of language impairment (Baddeley et al., 1998) that training targeting the core hypothesized deficit of verbal storage will enhance recall accuracy. However, support for this hypothesis in the analyses performed at the group level (LLA and comparison) was equivocal, as discussed above. The apparent inconsistency in the findings may reflect the fact that group assignment was based on different measures of language to the variables included in these exploratory individual difference analyses. The children in the LLA group were selected in this study on the basis of their performance on two verbal measures, a picture-word matching vocabulary test that required a pointing response (Dunn and Dunn, 2007) and a sentence repetition task. In contrast, verbal IQ is derived from a vocabulary test requiring the generation of definitions, and a similarities test involving comparison of the meanings of different words. It may therefore be the case that facility with the semantic aspects of language is a more critical determinant of response to training than the more phonologically based language abilities tapped by the screening tests.

Verbal IQ was both a positive and a negative predictor of children’s responses to working memory training. First, individuals with the lowest baseline verbal IQs made the greatest gains following training in verbal STM. Voluntary rehearsal is widely considered to commence on average at 7 years of age (Flavell et al., 1966; Gathercole et al., 1994), although in lower-achieving children such as the present sample this may be delayed. One possibility is that for these children, the repeated daily practice on multiple Cogmed tasks involving the retention of serial order of verbal material (digits and letters) may promote the development of simple rehearsal strategies, which in turn enhance verbal STM performance. Similar gains have certainly been demonstrated through explicit rehearsal strategy training in younger typically developing children (Johnston et al., 1987). This finding is encouraging, because verbal STM is often the weakest aspect of working memory in children with LLA. There may therefore be particular therapeutic value for working memory training in this population and, potentially, for children with more severe language learning deficits including SLI.

Second, individuals with higher baseline verbal IQs and non-word repetition scores made the greatest improvements on visuo-spatial working memory following training. These preliminary findings indicate that children’s responses to training may be directly modulated by their cognitive profiles, and that robust verbal abilities may be vital for the development of new strategies to meet the complex demands of visuo-spatial working memory tasks. For example, it may be easier for children with a strong facility for language to use verbal labels recode stimuli such as spatial locations or colors, providing them with additional ways of retaining the memory items. Consistent with this speculation, recent work has established that Cogmed training is associated with the development of efficient verbal grouping strategies (e.g., Dunning and Holmes, 2014).

This study is, to our knowledge, the first to evaluate whether responses to working memory training are modulated by children’s baseline cognitive skills. There are two key findings. First, working memory abilities do not appear to constrain responsiveness to training: the benefits of training for working memory in children with LLA accompanied by poor verbal STM and working memory were largely equivalent to those without language difficulties. Second, training appears to be particularly beneficial for verbal STM in individuals with low verbal abilities indexed by verbal IQ. Also, high verbal IQ may afford children opportunities to develop compensatory strategies through training. These results provide preliminary evidence that baseline cognitive abilities do indeed modulate the impact of working memory training, possibly in multiple ways.

## Conflict of Interest Statement

The authors declare that the research was conducted in the absence of any commercial or financial relationships that could be construed as a potential conflict of interest.
